# C2C12 Mouse Myoblasts Damage Induced by Oxidative Stress Is Alleviated by the Antioxidant Capacity of the Active Substance Phloretin

**DOI:** 10.3389/fcell.2020.541260

**Published:** 2020-09-11

**Authors:** Jie Li, Qing Yang, Lin Han, Chuanying Pan, Chuzhao Lei, Hong Chen, Xianyong Lan

**Affiliations:** ^1^Shaanxi Key Laboratory of Molecular Biology for Agriculture, College of Animal Science and Technology, Northwest A&F University, Yangling, China; ^2^School of Medicine, Sun Yat-sen University, Guangzhou, China; ^3^College of Food Science and Engineering, Northwest A&F University, Yangling, China

**Keywords:** phloretin, skeletal myopathies diseases, C2C12 myoblasts, AMPK, antioxidative

## Abstract

A new direction for the treatment of skeletal myopathies, which are mainly caused by abnormal mitochondrial metabolism, is the application of drugs and active substances to relieve oxidative stress in mitochondria. Phloretin, a dihydrochalcone active substance widely present in succulent fruits, has attracted attention for its strong antioxidant activity. This study aimed to investigate the potential antioxidant effects of phloretin and its potential mechanism of action in C2C12 mouse myoblasts. Under oxidative stress caused by 500 μmol/L H_2_O_2_, the addition of 10 μmol/L phloretin ameliorated the high level of reactive oxygen species, increased CuZn/Mn-dependent superoxide dismutase activities, and restored the loss of mitochondrial membrane potential. Additionally, apoptosis, necrocytosis, and the inhibition of cell proliferation caused by H_2_O_2_ stimulation were alleviated by phloretin. Moreover, phloretin significantly increased the expression of cyclin D1 and alleviated the stagnation trend of the G1 phase of cell proliferation caused by H_2_O_2_. Furthermore, the addition of phloretin simultaneously significantly increased the protein and mRNA expression of heme oxygenase-1 (HO-1) and alleviated the inhibitory phosphorylation of p-nuclear factor erythroid 2-related factor 2 (Nrf2), p-AMP-activated protein kinase (AMPK), and p-liver kinase B1 (LKB1) induced by H_2_O_2_. Moreover, the expression of nuclear Nrf2 was higher with phloretin treatment than without phloretin treatment. Overall, phloretin alleviated the proliferation inhibition and apoptosis induced by H_2_O_2_ and exerted antioxidant effects via the LKB1/AMPK/Nrf2/HO-1 pathway in C2C12 cells. These results provide insight for the application of phloretin to alleviate oxidative damage to muscle.

## Introduction

Skeletal myopathies, which are common in children and adolescents, are characterized by the inability of the skeletal muscle to tolerate fatigue. Such muscle damage is caused by abnormal energy supply, mainly via abnormal mitochondrial metabolism in muscle cells (Tarnopolsky, 2016). The most common cause of mitochondrial metabolic abnormalities is oxidative damage owing to the excess production of reactive oxygen species (ROS) in the mitochondrial respiratory chain (Brand, 2016). Angiotensin II, the active component of the renin-angiotensin-aldosterone system, increases mitochondrial ROS production in skeletal muscle (Semprun-Prieto et al., 2011; Sukhanov et al., 2011). Oxidative stress in skeletal muscle induces proteolysis and leads to atrophy (Leitner et al., 2017). Therefore, the application of drugs and bioactive compounds to relieve oxidative stress caused by ROS is a new direction for the treatment of muscle diseases (Moulin and Ferreiro, 2017).

Owing to the side effects of drug treatment, more people are currently considering the use of active substances, especially active substances derived from succulent fruits. Phloretin, a naturally occurring flavonoid that belongs to the dihydrochalcone chemical class, is present in juicy fruits, fruit peels, and plant roots, such as those of apple and pear plants (Wang et al., 2014; Li et al., 2018). Recently, various biological activities of phloretin have been reported, including anti-tumor (Wu et al., 2018; Choi, 2019), anti-inflammatory (Alsanea et al., 2017; Wang G. et al., 2018), and anti-oxidation properties (Ren et al., 2016; Yang et al., 2018; Zhang et al., 2019). Phloretin has been reported to remove excess ROS from several types of cells through the redox signaling pathways, such as nuclear factor-κB/mitogen-activated protein kinase (Huang et al., 2015) and liver kinase B1 (LKB1)/AMP-activated protein kinase (AMPK) pathways (Yang et al., 2018), to exert antioxidant activity. However, whether the antioxidant activity of phloretin is exerted in skeletal muscle cells, and can be further used for the treatment of muscle diseases, such as skeletal myopathies, are still unknown.

A typical cell model for muscle development and differentiation research *in vitro* are C2C12 mouse myoblasts, precursor cells in adult mouse skeletal muscle tissue that rebuild muscle tissue after trauma, with excellent ability for growth and differentiation (Sondag et al., 2014). Therefore, in this study, C2C12 mouse myoblasts and a typical oxidative stress modeling method, hydrogen peroxide stimulation, were used to explore whether phloretin can effectively relieve oxidative damage in skeletal muscle cells. Furthermore, the related mechanism was explored to provide a new theoretical basis for the diet-based treatment of muscle metabolic diseases.

## Materials and Methods

### Materials

A stock solution of phloretin (analytical grade, purity 98%) was purchased from Yuanye Biotech. Co. (Shanghai, China) and Compound C (MedChemExpress, United States), an inhibitor of AMPK, was prepared in dimethyl sulfoxide (DMSO). In all experiments, DMSO was diluted to less than 0.1% (w/v) by phosphate buffered saline (PBS) and further diluted in growth medium (Yang et al., 2018).

Antibodies, including anti-phospho-AMPKα (Thr172), anti-proliferating cell nuclear antigen (PCNA), anti-Cyclin D1, anti-GAPDH, and goat anti-rabbit and anti-mouse secondary antibody, were purchased from Cell Signaling Technology (Shanghai, China); anti-β-actin, anti-caspase 3, anti-caspase 9, and anti-Keap1 were obtained from Proteintech (Hubei, China); and anti-nuclear factor erythroid 2-related factor 2 (Nrf2), anti-phospho-Nrf2 (Ser40), anti-phospho-LKB1 (Ser428), and anti-heme oxygenase-1 (HO-1) were obtained from Santa Cruz Biotechnology (United States).

### Cell Culture and Oxidative Stress Modeling

In this study, the C2C12 mouse myoblast cell line was supplied by the Shanghai Cell Bank of the Chinese Academy of Sciences (Shanghai, China). Based on a previous study, C2C12 cells were cultured in Dulbecco’s modified Eagle’s growth medium containing 10% heat-inactivated fetal bovine serum and 1% penicillin/streptomycin, and maintained in a humidified atmosphere with 5% CO_2_ at 37°C (Zhang et al., 2018).

A previous study reported that the treatment of C2C12 cells with 200–1,000 μmol/L H_2_O_2_ causes oxidative stress in cells (Choi, 2018; Kim and Yi, 2018). Firstly, 500, 750, and 1,000 μmol/L H_2_O_2_ were added to C2C12 cells for 1 h in a preliminary experiment to stimulate oxidative stress. Secondly, based on cell morphology observation (Supplementary Figure S1), the final concentration of 500 μmol/L H_2_O_2_ was selected for subsequent experiments. Additionally, the 500 μmol/L H_2_O_2_ solution was generated as follows: the filtered 30% H_2_O_2_ solution was diluted with sterile PBS at a ratio of 1:199 and further diluted in growth medium, until the 500 μmol/L final concentration was achieved.

The four groups in this study were control group (treated with diluted DMSO), H_2_O_2_-stimulated group, phloretin-treated group, and 10 μmol/L phloretin and H_2_O_2_ co-treatment group.

### Determination of ROS Production

C2C12 cells were inoculated into 12-well plates. When the density of cells reached 60–70%, the cells were washed with PBS. The experimental groups for ROS detection were as follows: control group treated with diluted DMSO, H_2_O_2_-stimulated group, and 10 μmol/L phloretin treatment followed by co-incubation with 500 μmol/L H_2_O_2_ in the final hour group. All treatments lasted for 12 h, after which the medium was discarded and the ROS detection probe 2′-7′dichlorofluorescin diacetate (10 μmol/mL) was added at 37°C. Subsequent processing steps were fully consistent with the instructions of the ROS Assay Kit (Beyotime Biotechnology, Shanghai, China). There were three biological replicates in each group, and each biological replicate was repeated six times.

### Cell Counting Kit-8 (CCK8) Assay for Cell Activity and Cell Proliferation

To detect cell activity, C2C12 cells were seeded into 96-well plates. When the density of cells reached 40–60%, the specified dose of phloretin or diluted DMSO was added to the medium. Afterward, the cells in each well were treated according to instructions of CCK8 with WST-8 (Beyotime Biotechnology, Shanghai, China). After incubation for 24 h, cells were washed once with sterile PBS, and 10 μL of CCK8 staining solution and 100 μL of serum-free medium were added to each well. Incubation continued for 2–4 h in the dark, and the absorbance of WST-8, which is directly proportional to the viability of C2C12 cells, was measured at 450 nm.

### Measurement of CuZn/Mn-Dependent Superoxide Dismutase (SOD) Activity and Mitochondrial Membrane Potential (MMP)

The CuZn/Mn-SOD activities in C2C12 cells were measured according to the instructions of the CuZn/Mn-SOD Assay Kit (Beyotime Biotechnology, Shanghai, China). The results were normalized with respect to the control group, and each treatment had three independent replicates (Han et al., 2020).

The MMP (ΔΨm) was detected using a fluorescent probe: 5,5′,6,6′-tetrachloro-1,1′,3,3′-tetraethyl benzimidazolyl carbocyanine iodide (JC-1; Beyotime Biotechnology, Shanghai, China). The increase in the number of JC-1 monomers and decrease in the number of J-aggregates, which are always accompanied by mitochondrial depolarization, were detected by a multi-detection microplate reader, following the manufacturer’s instructions.

### Annexin-V-Fluorescein Isothiocyanate (FITC) and Propidium Iodide (PI) Double Staining Assay

Based on the instructions of the Annexin V-FITC Apoptosis Detection Kit (Beyotime Biotechnology, Shanghai, China), the cells were harvested, washed with PBS, and resuspended in a 500 μL of 1× binding buffer (5 μL of annexin-V-FITC and 5 μL of PI, Biobox). Subsequently, cells were incubated for 30 min at 20–25°C in the dark and analyzed by flow cytometry (BD FACSAria III, BD Biosciences). The data were analyzed using the FCS Express 5.0 Software (De Novo Software).

### Cell Cycle Distribution

The cell cycle analysis method was based on a previous study (Chen et al., 2018). C2C12 cells, which were untreated or treated with 10 μmol/L phloretin for 24 h and stimulated with 500 μmol/L H_2_O_2_ for 1 h, were washed with PBS and digested with trypsin. Afterward, cells were centrifuged at 2,000 × *g* for 5 min and the collected cells were fixed using 1 mL of 70% pre-cooled ethanol diluted with PBS for 1 h at −20°C. After incubation with 50 μg/mL PI (Sigma-Aldrich, St. Louis, MI, United States), cells were analyzed by flow cytometry (BD FACSAria III, BD Biosciences) and the data were analyzed using the FCS Express 5.0 Software (De Novo Software) (Chen et al., 2018).

### Isolation of Total RNA and Quantitative RT PCR Analysis

Following the manufacturer’s instructions, total cellular RNA was isolated using TRIzol reagent (Life Technologies, Carlsbad, CA, United States). Then, the cDNA Synthesis Kit (Life Technologies, Shanghai, China) was used for cDNA synthesis from isolated total RNA. Primers for cell proliferation and apoptosis marker genes (Table 1) were used for quantitative RT-PCR (qRT-PCR), and TB Green Master Mix (TaKaRa) and ABI 7300 software (Applied Biosystems, United States) were applied for the quantification of the results. Each treatment group contained three biological replicates, and each biological replicate consisted of three technical replicates. The 2^–ΔΔCT^ method was selected to analyze relative fold changes of candidate genes (Zhang et al., 2016) and *β-actin* was used for normalization.

**TABLE 1:** The primers used for qRT-PCR.

Genes	Forward primers (5′ to 3′)	Reverse primers (5′ to 3′)
SOD1	GCTTCTCGTCTTGCTCTCTC	GCTGGCCTTCAGTTAATCCT
SOD2	GAACGGCCGTGTTCTGAG	GGGAGGCTGTGCTTGTG
GPx	CCTGGCCGGGTTTGTTC	ATGGTGAGGGCTCCATACT
CAT	CCGCAATCCTACACCATGT	TGGTCAGGACATCAGGTCT
HO-1	AGCCTGAATCGAGCAGAAC	TCAAGGCCTCAGACAAATCC
Cyclin D1	TCTACACTGACAACTCTATCCG	TAGCAGGAGAGGAAGTTGTTGG
Cyclin E	GTGGCTCCGACCTTTCAGTC	CACAGTCTTGTCAATCTTGGCA
Bax	TGAAGACAGGGGCCTTTTTG	AATTCGCCGGAGACACTCG
Bcl2	ATGCCTTTGTGGAACTATATGGC	GGTATGCACCCAGAGTGATGC
P53	GCGTAAACGCTTCGAGATGTT	TTTTTATGGCGGGAAGTAGACTG
P21	CCTGGTGATGTCCGACCTG	CCATGAGCGCATCGCAATC
Caspase3	AGTTCCCGGGTGCTGTCTAT	GCCATGGTCTTTCTGCTCAC
Caspase9	CCACTGCCTCATCATCAAC	TGTGCCATCTCCATCAAA
GAPDH	AGGTCGGTGTGAACGGATTTG	TGTAGACCATGTAGTTGAGGTCA
β-actin	CCTAAGGCCAACCGTGAAA	TGGTACGACCAGAGGCATA

### Western Blot Analysis

Protein was extracted from harvested cells using radioimmunoprecipitation assay buffer (Beyotime Biotechnology, Shanghai, China) based on a previously established procedure (Chen et al., 2018). After quantification by the BCA Protein Assay Kit (TaKaRa, T9300A), equal amounts of protein (30–50 μg) were separated by 10% sodium dodecyl sulfate-polyacrylamide gel electrophoresis and transferred onto polyvinylidene fluoride membranes (Millipore, Germany). Membranes were blocked with 4% bovine serum albumin and incubated with a specific primary antibody overnight at 4°C and incubated with a secondary antibody for 1 h at room temperature. Bands of proteins were visualized using a chemiluminescence assay system on a ChemiDoc Touch Imaging System (Bio-Rad). Finally, the relative densities of the individual bands were analyzed using the ImageJ software. GAPDH and β-actin were used as loading controls.

### Statistical Analysis

All statistical analyses were performed using SPSS 18.0. Statistical analysis was performed using a one-way analysis of variance to compare the means among different groups with normally distributed data (Yang et al., 2018). For data that did not follow a normal distribution, the homogeneity of variances was analyzed using the non-parametric Kruskal–Wallis test (Chen et al., 2018). All data are presented as the mean ± SEM, and *P* < 0.05 was considered statistically significant.

## Results

### The Phloretin Treatment Concentration and Oxidative Stress Model Were Established

**FIGURE 1:**
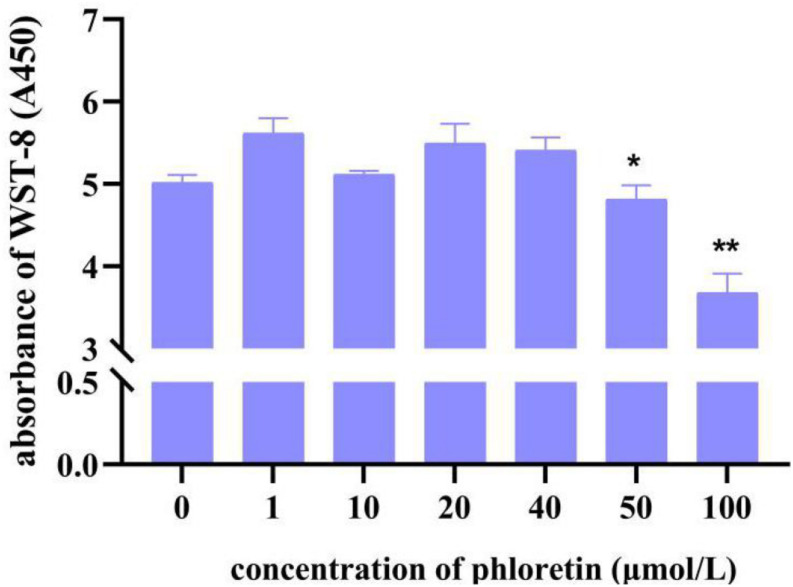
C2C12 cell viability of different concentrations of phloretin using the CCK8 assay. * and ** Mean significant difference refer to *P* < 0.05 and *P* < 0.01, respectively.

**FIGURE 2:**
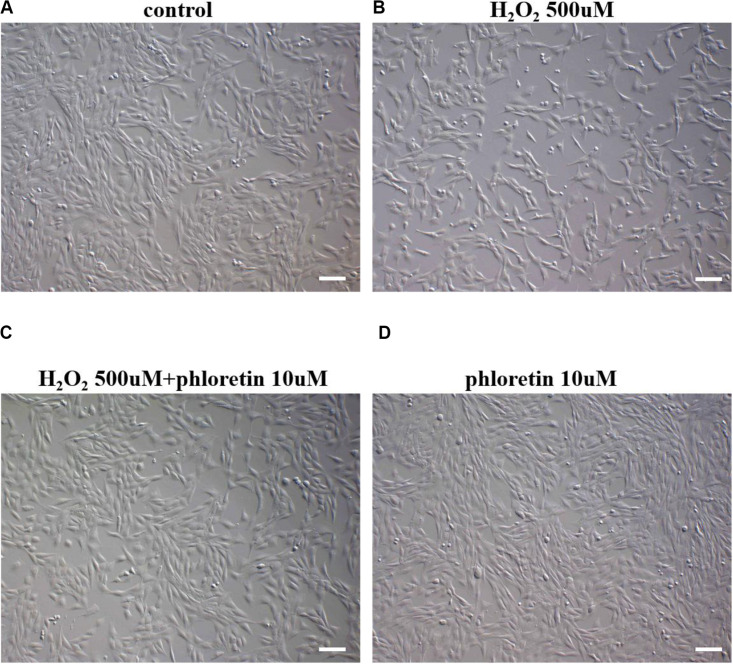
Morphology and numbers of C2C12 cells after supplementation with phloretin or H_2_O_2_ in different groups. When the density of cells reached approximately 30%, diluted DMSO was added to the **(A)** control group and **(B)** H_2_O_2_-stimulated group, whereas 10 μmol/L phloretin was added to the **(C)** co-induction group and **(D)** phloretin group for 24 h. For the final 1 h, 500 μmol/L H_2_O_2_ was added to **(B,C)**. Scale bar = 100 μm.

The results of cell viability assay (Figure 1) and cell morphology observation (Supplementary Figure S1) showed that phloretin had no toxic effects on cells when the concentration is less than 50 μmol/L. However, various concentrations of H_2_O_2_, including 500, 750, and 1,000 μmol/L, significantly altered cell morphology and reduced the number of living cells, indicating the success of the oxidative stress modeling (Figure 2 and Supplementary Figure S1A).

The morphology of C2C12 cells treated with three concentrations of phloretin, 10, 20, and 40 μmol/L, and co-treated with 500, 750, and 1,000 μmol/L of H_2_O_2_ were observed. When the concentration of hydrogen peroxide was 1000 μmol/L, no concentration of phloretin could restore the morphology of the cells to normal (Supplementary Figure S1B); thus, the H_2_O_2_ concentrations of 500 and 750 μmol/L were selected for further experiments.

**FIGURE 3:**
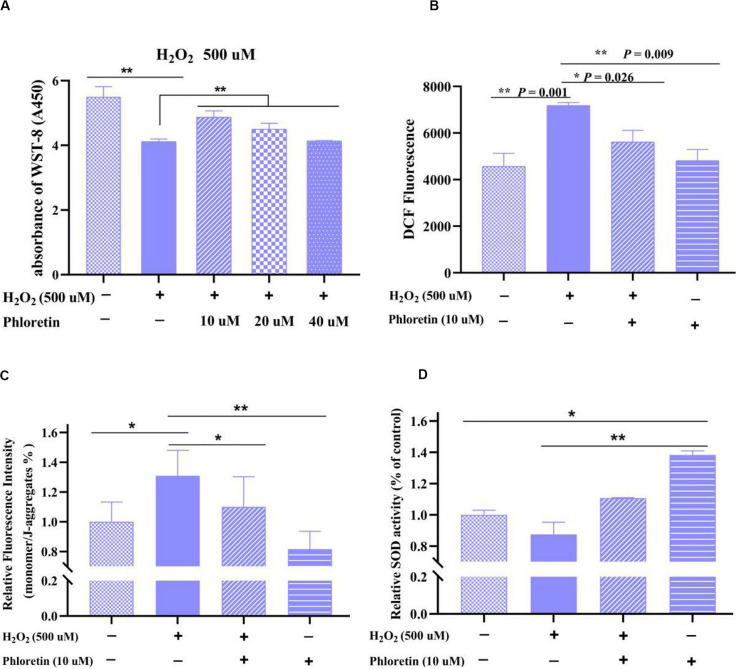
Detection of **(A)** cell viability, **(B)** ROS level, **(C)** mitochondrial membrane potential, and **(D)** superoxide dismutase activity of C2C12 cells in different groups. ** and * represent great significant difference (*P* < 0.01) and significant difference (*P* < 0.05), respectively.

Based on the CCK8 assay results, in the co-treatment groups, the highest cell viability was detected in 10 μmol/L phloretin among 500 μmol/L H_2_O_2_ treated groups (Figure 3A), and 40 μmol/L phloretin among 750 μmol/L H_2_O_2_ treated groups (Supplementary Figure S2). Hence, the combination of 10 μmol/L phloretin and 500 μmol/L H_2_O_2_ was selected for subsequent experiments, whereas the combination of 40 μmol/L phloretin and 750 μmol/L H_2_O_2_, was used in a supplementary group for more pronounced results.

### Phloretin Attenuated Oxidative Stress and Restored the MMP Loss in C2C12 Cells

Based on cell morphology observation, the damage to cell morphology by H_2_O_2_ was alleviated by phloretin (Figures 2B,C). Furthermore, based on the results of CCK8 and ROS detection, ROS production in the phloretin and H_2_O_2_ co-treatment group was significantly lower than those in the H_2_O_2_ stimulation group, while trends of cell viability were contrary (Figures 3A,B).

In normal cells, the electron transport chain in mitochondria is one of the main sources of ROS and the excessive generation of ROS by oxidative stress causes the MMP level to drop, which seriously disrupts the function of cells (Brand, 2016). Herein, consistent with these previous findings (Yang et al., 2018), the MMP in H_2_O_2_-stimulated cells was significantly lower than that in control. However, this decrease was significantly reversed by 10 μmol/L phloretin preconditioning, which revealed that phloretin restores the loss of MMP caused by excessive ROS in C2C12 cells (Figure 3C).

### Phloretin Increased the Activity and Gene Expression of Antioxidant Enzymes

The antioxidant enzymes in the body, such as SOD, play key roles in the removal of excessive ROS to relieve oxidative stress. In this study, co-treatment with phloretin and H_2_O_2_ significantly reversed the decrease of CuZn/Mn-dependent SOD enzyme activity caused by H_2_O_2_ (Figure 3D). Additionally, the activity of SOD in C2C12 cells treated with phloretin was significantly higher than that in control cells (Figure 3D).

**FIGURE 4:**
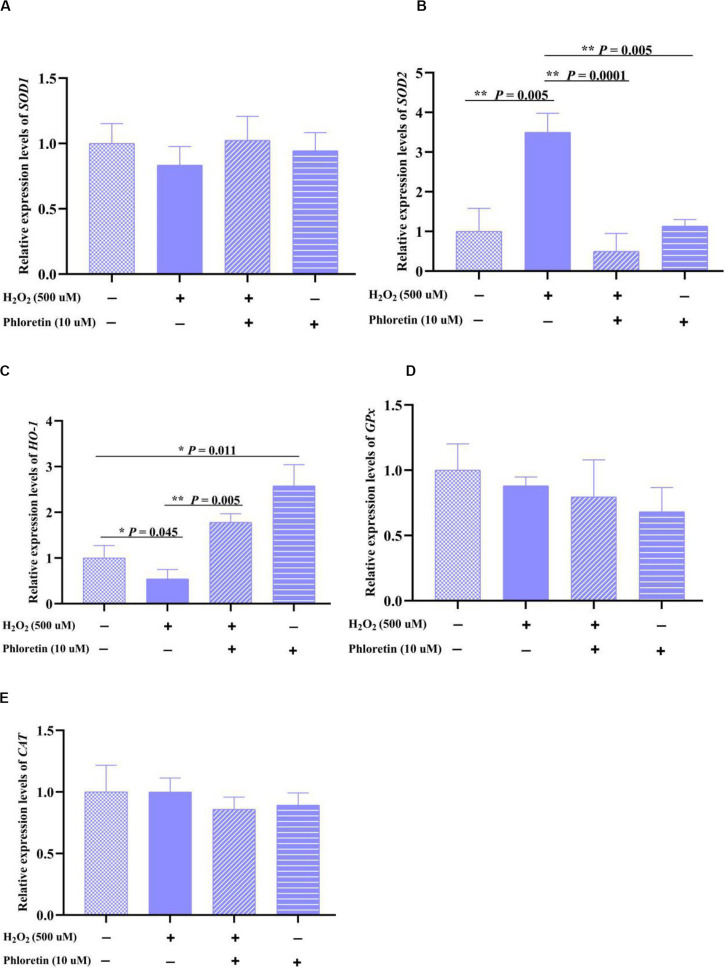
Relative expression levels of oxidation-reduction enzyme-encoding genes, **(A)** SOD1, **(B)** SOD2, **(C)** HO-1, **(D)** Gpx and **(E)** CAT in different treatment groups. ** and * represent great significant difference (*P* < 0.01) and significant difference (*P* < 0.05), respectively.

Correspondingly, changes in the transcription levels of the coding genes *SOD1* and *SOD2* were detected. The trend of *SOD1* expression in each group was consistent with that of SOD enzyme activity, but there was no significant difference in *SOD1* expression among groups; contrarily, the trend of *SOD2* expression was the opposite to that of SOD enzyme activity (Figures 4A,B). This revealed that phloretin does not increase SOD enzyme activity via the promotion of SOD expression.

The expression of other oxidation-reduction enzyme-encoding genes, *GPx*, *CAT*, and *HO-1*, was also explored. Phloretin significantly alleviated the decrease in *HO-1* expression caused by H_2_O_2_ stimulation (Figure 4C), whereas it did not significantly affect the expression of *GPx* and *CAT* (Figures 4D,E).

### Phloretin Alleviated Cell Proliferation Inhibition Under Oxidative Stress

**FIGURE 5:**
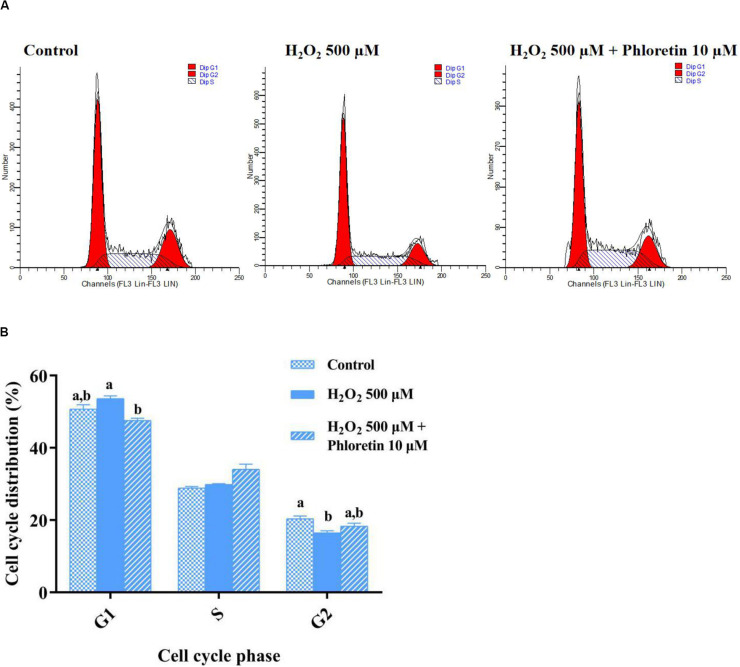
Effect of phloretin on the cell cycle distribution of C2C12 cells. **(A)** Representative diagram of cell cycle with different treatment. **(B)** Histogram of each cell cycle in different groups. a and b represent significant difference with *P* < 0.05.

In addition to the attenuation of oxidative stress, in the phloretin and H_2_O_2_ co-treatment group, the cell proliferation inhibition caused by H_2_O_2_ was alleviated (Figures 2B,C). To uncover the process involved in the alleviation of cell proliferation inhibition by phloretin, cell cycle analysis was performed. After pre-protective treatment with 10 μmol/L phloretin for 24 h, the C2C12 cells were stimulated with 500 μmol/L H_2_O_2_ for 1 h, and the cell cycle was analyzed. There was no statistically significant difference in the proportion of cells in the S phase among groups (Figure 5 and Table 2). However, the difference in the proportion of cells in the G1 phase among groups revealed that H_2_O_2_ stimulation tended to arrest cell proliferation in the G1 phase, and this arrest was significantly alleviated with the addition of phloretin (Figure 5).

**TABLE 2:** Cell cycle distributions of C2C12 cells.

Groups	G1 (%)	S (%)	G2 (%)	S+G2 (%)
Control	50.747 ± 1.166^a,b^	28.890 ± 0.395	20.363 ± 0.783^a^	49.253 ± 1.162^a,b^
H_2_O_2_-500 μM	53.635 ± 0.715^a^	29.870 ± 0.180	16.490 ± 0.530^b^	46.360 ± 0.710^b^
Phloretin 10 μM + H_2_O_2_ 500 μM	47.575 ± 0.585^b^	34.085 ± 1.365	18.335 ± 0.785^a,b^	52.420 ± 0.580^a^

*“a” and “b” represent significant difference with P < 0.05.*

### Phloretin Altered the Expression of Cell Cycle Proteins

**FIGURE 6:**
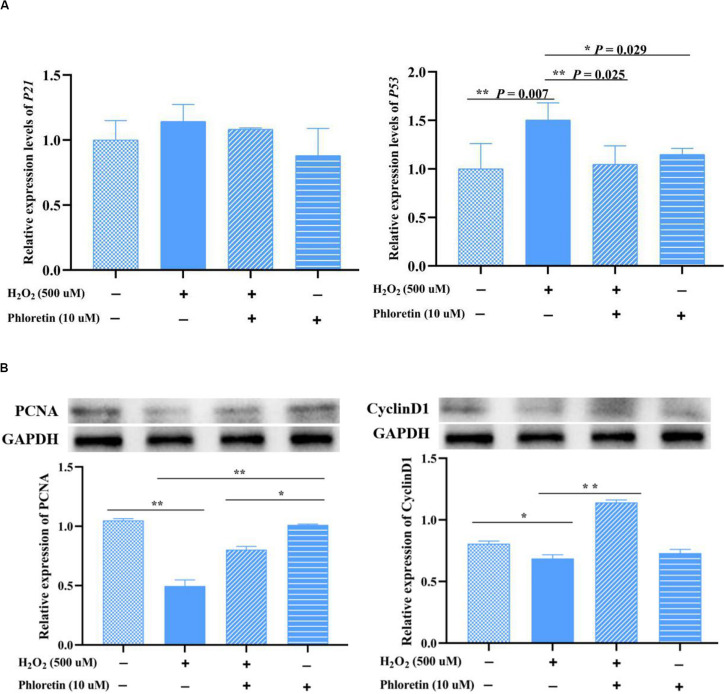
Effect of phloretin and H_2_O_2_ on the expression of cell proliferation genes at the transcriptional **(A)** and translational **(B)** levels. ** and * represent great significant difference (*P* < 0.01) and significant difference (*P* < 0.05), respectively.

To further understand the cell cycle was modulated by phloretin, the expression levels of cell cycle proteins and their coding genes were investigated by western blotting and qRT-PCR, respectively. The protein expressions of cyclin D1 and PCNA were significantly higher in the phloretin and H_2_O_2_ co-treatment group than H_2_O_2_ stimulation group, which showed that phloretin alleviates proliferation inhibition under oxidative stress (Figure 6B). However, no significant differences were detected among groups in the transcription levels of the genes coding these proteins. As the main inhibitors upstream of *cyclinD*, the expression levels of *P21* and *P53* in the phloretin and H_2_O_2_ co-treatment group were lower than those in the H_2_O_2_ stimulation group, and *P53* expressions were significantly different (Figure 6A and Supplementary Figure S3). This indicated that the inhibition of *P21* and *P53* by phloretin promotes the expression of the cell cycle protein cyclin D1, which plays a key role in the G1 phase.

### Phloretin Reduced the Percentages of H_2_O_2_-Induced Apoptotic and Necrotic Cells

**FIGURE 7:**
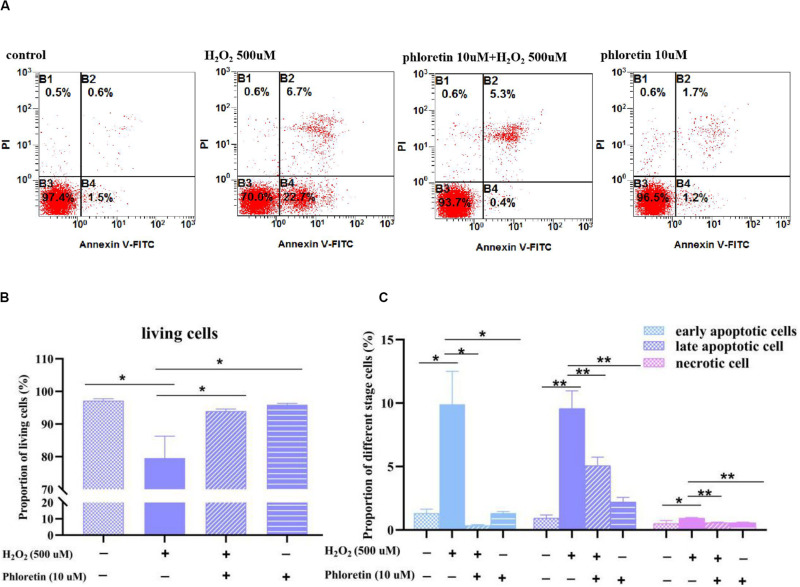
Effect of phloretin and H_2_O_2_ on the percentages of apoptotic and necrotic cells. **(A)** Representative diagram of Annexin-V-FITC&PI with different treatment. Histogram of percentages of **(B)** living cells, **(C)** apoptotic and necrotic cells in different groups. * and ** represent significant difference with *P* < 0.05 and *P* < 0.01, respectively.

As oxidative stress causes apoptosis and necrosis, flow cytometry was used to explore apoptotic and necrotic cells among different groups. The percentages of necrotic and early and late apoptotic cells were markedly higher, whereas the percentage of living cells was significantly lower in the H_2_O_2_ stimulation group than in the control group. However, in the phloretin and H_2_O_2_ co-treatment group, phloretin significantly alleviated the percentages of apoptotic and necrotic cells (Figure 7).

### Phloretin Alleviated the H_2_O_2_-Induced Expression of Apoptosis-Related Genes and Proteins

**FIGURE 8:**
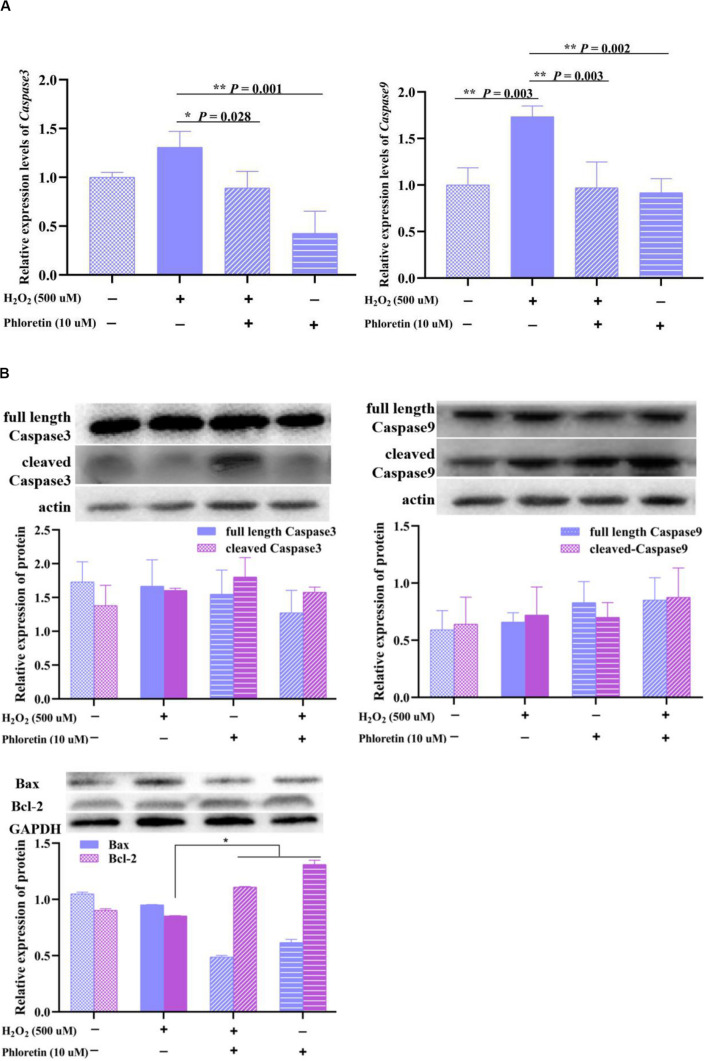
Effect of phloretin and H_2_O_2_ on C2C12 apoptosis. **(A)** q-RT PCR assay, **(B)** western blot analysis.

To explore the process involved in apoptosis and necrosis due to oxidative stress, the expressions of related genes and proteins were detected. qRT-PCR showed that the expression levels of *caspase3* and *caspase9* were significantly lower with phloretin and H_2_O_2_ co-treatment than H_2_O_2_ stimulation (Figure 8A). However, phloretin was not involved in the transcriptional and translational regulation of *caspase3*, *caspase9*, and their activated forms (Figure 8B). Nevertheless, the decline in the expression of Bcl-2 induced by H_2_O_2_ was alleviated by phloretin (Figure 8B).

These results indicated that phloretin exerts antioxidant functions by the transcriptional and translational regulation of cell proliferation and apoptosis.

### Phloretin Exerted Antioxidant Effects via the AMPK/Nrf2/HO-1 Pathway

**FIGURE 9:**
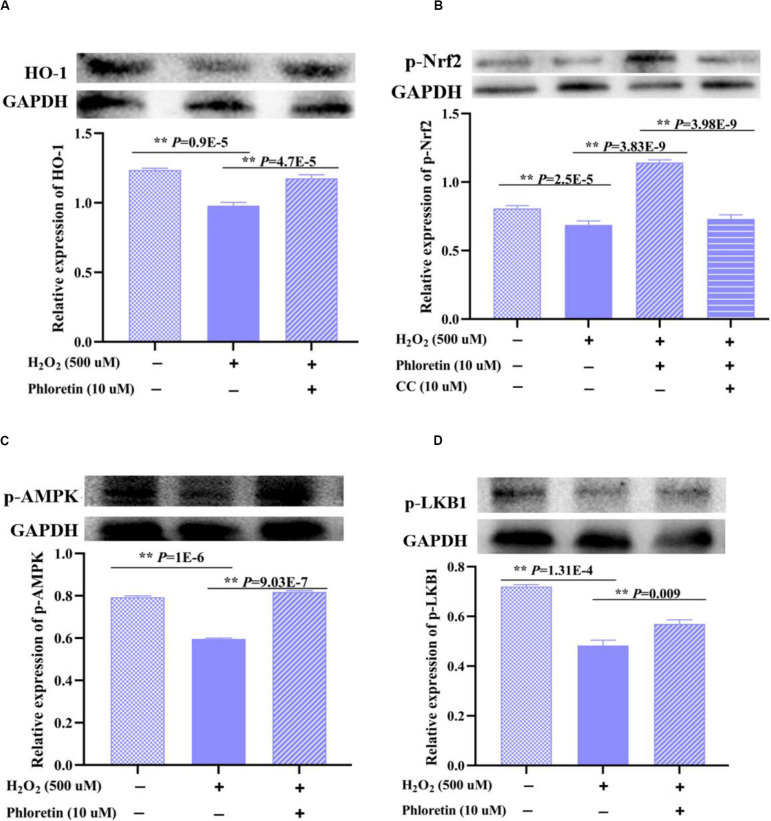
Effect of phloretin on LKB1/AMPK/Nrf2/HO-1 activity in C2C12 cells. Cells at a density of 70% were treated (or not treated) with 10 μmol/L phloretin and 500 μmol/L H_2_O_2_. **(A)** Protein expression of HO-1 and phosphorylation levels of **(B)** p-Nrf2, **(C)** p-AMPK, and **(D)** p-LKB1 were detected. Cells were cultured with compound C (10 μmol/L), an inhibitor of AMPK, for 3 h in **(B)**.

HO-1, an enzyme with extremely strong antioxidant activity that plays a vital role in the alleviation of increased intercellular ROS levels, was detected in different treatment groups. The expression of HO-1 in the H_2_O_2_ stimulation group was significantly lower than that in the control group. In contrast, with the addition of phloretin, the expression of HO-1 was promoted (Figure 9A), consistent with the changes in the transcription level of *HO-1* (Figure 4C).

Subsequently, Nrf2, an upstream regulator of HO-1, was examined. The expression trend of the phosphorylated Nrf2 protein was consistent with that of the HO-1 protein among groups (Figure 9B).

There are various upstream regulators of Nrf2, such as AMPK (Nguyen et al., 2015). After treatment with phloretin and the AMPK protein inhibitor compound C, phosphorylated Nrf2 protein was significantly decreased compared to that in the cells treated with phloretin only (Figure 9B). Furthermore, the addition of phloretin significantly alleviated the H_2_O_2_-induced inhibition of AMPK phosphorylation (Figure 9C), indicating that phloretin exerts antioxidant effects via the activation of the AMPK/Nrf2/HO-1 pathway.

As a vital regulatory protein of AMPK, the expression trend of p-LKB1 was consistent with that of p-AMPK among groups (Figure 9D).

### Phloretin Increased the Expression of Nuclear Nrf2

**FIGURE 10:**
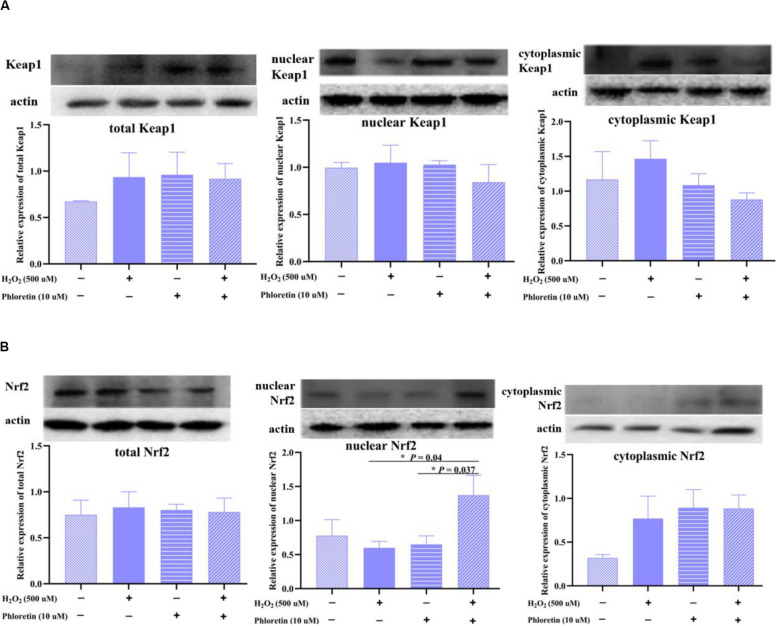
Effect of phloretin on the expression of **(A)** Keap1 protein and **(B)** Nrf2 protein in different components of cells. * Mean significant difference (*P* < 0.05).

Nrf2 is sequestered by cytoplasmic Keap1 and targeted for proteasomal degradation under basal conditions; under oxidative stress, Nrf2 detaches from Keap1 and translocates to the nucleus (Bellezza et al., 2018). In this study, although no significant difference existed in total and cytoplasmic Nrf2 and Keap1 proteins expression among groups (Figure 10A). The expression of endonuclear Nrf2 in the H_2_O_2_ stimulation group was lower than that in the control group. Additionally, the expression of endonuclear Nrf2 in the phloretin and H_2_O_2_ co-treatment group increased significantly compared with H_2_O_2_ stimulation group (Figure 10B), which was in accordance with the expression trend of the phosphorylated Nrf2 protein.

**FIGURE 11:**
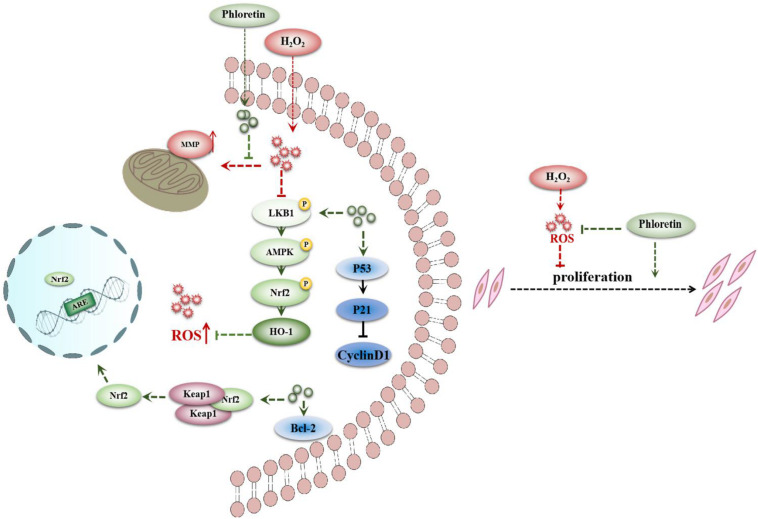
Proposed signaling pathways for phloretin to ameliorate oxidative stress in C2C12 myoblasts.

The above results indicated that phloretin alleviates the proliferation inhibition and apoptosis of C2C12 cells induced by H_2_O_2_ and exerts antioxidant effects via the LKB1/AMPK/Nrf2/HO-1 pathway in C2C12 cells (Figure 11).

## Discussion

As a chalcone compound, phloretin is present in various succulent fruits. Studies have shown that phloretin relieves oxidative damage in certain types of cells, such as myocardial (Vineetha et al., 2015) and smooth muscle (Wang et al., 2015) cells. To investigate whether its effects are extensive, the effects of phloretin on oxidative stress in skeletal muscle cells was explored. In contrast to the promotion of the activity of caspase 3 in myocardial cells (Vineetha et al., 2015), phloretin was not involved in the expression of caspases in this study. It is speculated that these differences may be caused by different cell types or different phloretin concentrations. Moreover, although phloretin promoted C2C12 cells proliferation in this study, phloretin has been shown to inhibit abnormal cell proliferation in rat aortic smooth muscle cells (Wang et al., 2015). However, consistent with our results, the oxidative stress caused by H_2_O_2_ has been shown to inhibit cell proliferation, and phloretin attenuated this inhibition via the promotion of the expression of cell proliferation-associated proteins (Dimozi et al., 2015). Furthermore, phloretin ameliorates mitochondrial dysfunction in cardiomyoblasts (Vineetha et al., 2015). This is consistent with the results of this study and reflects the consistency and breadth of the anti-oxidation effect of phloretin.

Oxidative stress can cause abnormalities in the mitochondrial oxidative respiratory chain, which in turn, can aggravate the occurrence of muscle metabolic diseases. In this study, phloretin restored the loss of MMP and significantly decreased the level of excessive ROS in C2C12 cells, which is beneficial to maintain a steady electron transport chain in mitochondria. Clinically, skeletal myopathies mainly manifest as a progressive aggravation of skeletal muscle atrophy and weakness, with varying degrees and distribution. The current drug treatment for muscle atrophy is mainly focused on improvement of appetite, modulation of inflammation, and interference with anabolic and catabolic reactions using candidate medications, such as thalidomide (Cao et al., 2018). However, no medication has been shown to be effective in clinical trials so far. The use of a phloretin-based therapeutic regimen will likely be safe and reliable for the treatment of muscular atrophy. In skeletal muscle and C2C12 myoblasts, in addition to phloretin, other plant-derived bioactive substances abundant in fruits, such as myricitrin (Ahangarpour et al., 2018) and resveratrol (Haramizu et al., 2017), may exert antioxidant effects. Currently, various studies have linked healthy foods to the alleviation of degenerative diseases (Panche et al., 2016). However, the potential interactions among different antioxidant bioactive substances in a single type of cell or tissue are worth further exploration to provide insight into appropriate fruit and dietary combinations.

Furthermore, here, phloretin inhibited oxidation via the regulation of the LKB1/AMPK/Nrf2/HO-1 pathway. Phloretin has also been identified to exert antioxidant effects via the Nrf2 pathway in other cells, such as human umbilical vein endothelial cells (Yang et al., 2018). Nrf2, a key nuclear transcription factor, is a core regulator in the oxidative stress pathway (Chen and Maltagliati, 2018). Its upstream regulatory protein, AMPK, plays a major role in the maintenance of the intracellular energy balance and regulation of whole-body energy metabolism (Carling, 2017). Hence, the AMPK-Nrf2-dependent pathway is involved in the antioxidant and anti-inflammatory functions of other plant-derived bioactive substances, including quercetin (Wang D. et al., 2018), anthocyanins (Ho et al., 2017), procyanidin (Lu et al., 2018), salvianolic acid C (Song et al., 2018), and fortunellin (Zhao et al., 2017). For instance, phloretin may prevent diabetic cardiomyopathy via the dissociation of the Keap1/Nrf2 complex and inhibition of oxidative stress (Ying et al., 2018). In contrast to these studies, this study revealed a more comprehensive signaling pathway for the role of phloretin in oxidative stress regulation.

Additionally, previous intervention study has examined the effect of fresh apple consumption on oxidative markers in humans. The daily intake of fresh apple at 2 g/kg body weight for 30 days effectively increases the levels of antioxidant enzymes in tested participants (Avci et al., 2007). Therefore, it is speculated that a patient with muscle atrophy can effectively reduce oxidative stress by the ingestion of approximately two normal size apples per day (Hyson, 2011; Tu et al., 2017). For patients who cannot consume apples, the direct intake of pure phloretin products with liquid food or the consumption of freshly squeezed apple juice is recommended. Regardless of the method of intake, the intake of phloretin from succulent fruits undoubtedly provides a healthy and feasible treatment for patients with muscle diseases. However, the antioxidant mechanism of phloretin in skeletal muscle cells needs further validation *in vivo*.

## Conclusion

Phloretin mitigates oxidative damage in C2C12 cells by the alleviation of proliferation inhibition and apoptosis via the AMPK/Nrf2/HO-1 signaling pathway. The results of this study provide a strong scientific basis for the proper application of phloretin to promote the intake of healthy dietary combinations for treating skeletal myopathies.

## Data Availability Statement

All datasets generated for this study are included in the article/Supplementary Material.

## Author Contributions

JL: writing – original draft and validation. QY and LH: validation. CP: supervision, writing – review and editing. CL and HC: writing – review and editing. XL: supervision and project administration. All authors read and approved the final manuscript.

## Conflict of Interest

The authors declare that the research was conducted in the absence of any commercial or financial relationships that could be construed as a potential conflict of interest.

## Supplementary Material

FIGURE S1 Morphology and numbers of C2C12 cells after supplementation of phloretin and H_2_O_2_ with various concentrations in different groups. Scale bar = 100 μm. “^∗^” Means significant difference with *P* < 0.05.

FIGURE S2 Ros level of C2C12 cells under induction with 750 μmol/L H_2_O_2_ in different groups. “^∗∗^” and “^∗^” represent great significant difference (*P* < 0.01) and significant difference (*P* < 0.05), respectively.

FIGURE S3 The relative expression levels of *P21* and *P53* genes in different treatment groups (under induction with 750 μmol/L H_2_O_2_). “^∗∗^” and “^∗^” represent great significant difference (*P* < 0.01) and significant difference (*P* < 0.05), respectively.
