# Supplementary material for: C2C12 Mouse Myoblasts Damage Induced by Oxidative Stress Is Alleviated by the Antioxidant Capacity of the Active Substance Phloretin
Source: Front Cell Dev Biol. 2020 Sep 11;8:541260. doi: 10.3389/fcell.2020.541260 (PMC7516399; doi:10.3389/fcell.2020.541260)
Supplement: FIGURE S1 — Morphology and numbers of C2C12 cells after supplementation of phloretin and H2O2 with various concentrations in different groups. Scale bar = 100 μm. “∗” Means significant difference with P < 0.05. [file Image_1.pdf]

Supplementary Figures

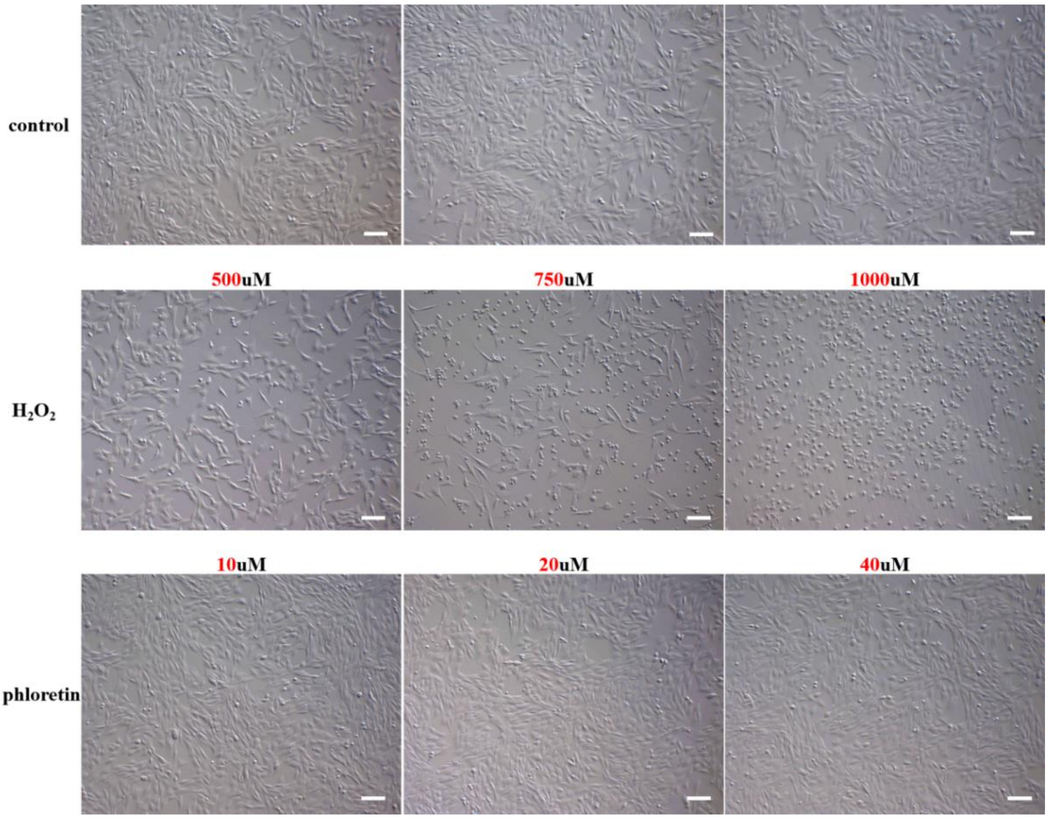

(B)

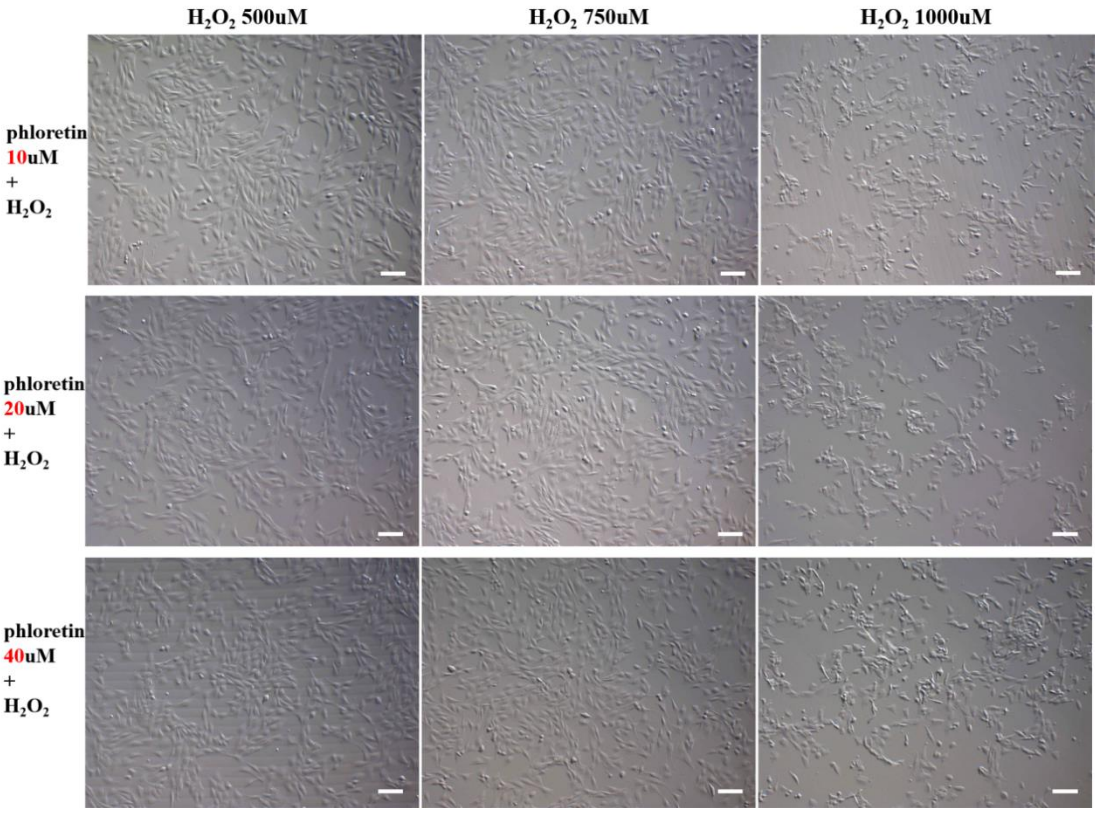

Figure S1. Morphology and numbers of C2C12 cells after supplementation of phloretin and H_2O_2 with various concentrations in different groups. Note: scale bar＝100 µm. "*" means significant difference with P＜0.05.

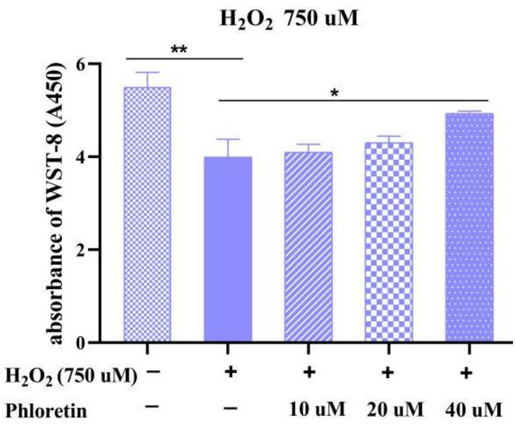

Figure S2. ROS level of C2C12 cells under induction with 750 µmol/L H_2O_2 in different groups. Note: "**" "*" represent great significant difference (P＜0.01) and significant difference (P＜0.05), respectively.

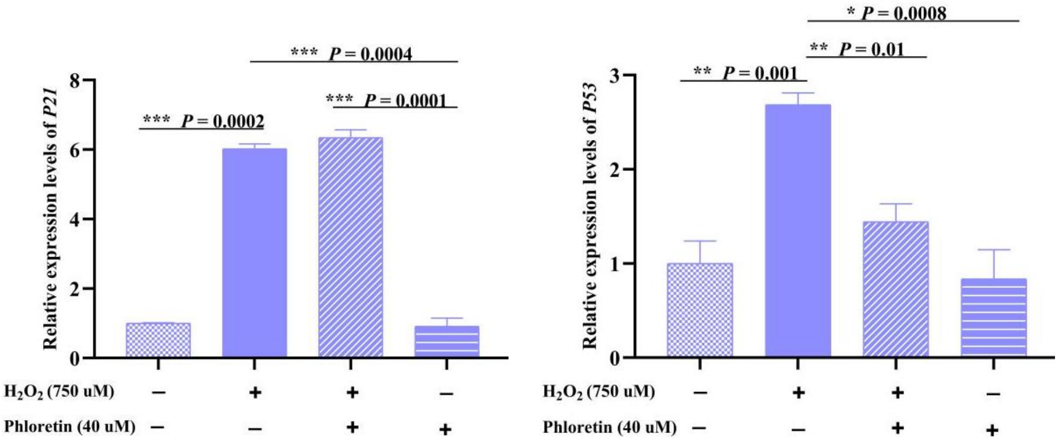

Figure S3. The relative expression levels of *P21* and *P53* genes in different treatment groups (under induction with 750 µmol/L H_2O_2). Note: "**" "*" represent great significant difference (P＜0.01) and significant difference (P＜0.05), respectively.
